# Relationship between serum tumor markers and Anaplastic Lymphoma Kinase mutations in stage IV lung adenocarcinoma in Hubei province, Central China

**DOI:** 10.1002/jcla.23027

**Published:** 2019-09-06

**Authors:** Qi Tan, Qi Huang, GuanZhou Ma, Zhilei LV, PeiYuan Mei, KaiMin Mao, Feng Wu, Yang Jin

**Affiliations:** ^1^ Department of Respiratory and Critical Care Medicine NHC Key Laboratory of Pulmonary Diseases Union Hospital Tongji Medical College Huazhong University of Science and Technology Wuhan China; ^2^ Department of Thoracic Surgery Union Hospital Tongji Medical College Huazhong University of Science and Technology Wuhan China

**Keywords:** anaplastic lymphoma kinase, carcinoembryonic antigen, CYFRA21‐1, neuron‐specific enolase, squamous cell carcinoma antigen

## Abstract

**Objective:**

The aim of this study was to explore the predictive value of carcinoembryonic antigen (CEA), squamous cell carcinoma antigen (SCCAg), and neuron‐specific enolase (NSE) in the prediction of anaplastic lymphoma kinase (ALK) mutations in advance stage non‐small cell lung cancer (NSCLC).

**Subjects and Methods:**

A total of 482 cases with untreated lung adenocarcinoma were retrospectively reviewed. Finally, 72 patients with stage IV were enrolled because of intact data of the detection of ALK rearrangement and serum tumor markers, as well they have not received any previous anticancer therapy. We used the one‐way ANOVA analysis, correlation analysis, and multiple logistic regression analysis to evaluate the relationship between the level of serum tumor markers and ALK mutations.

**Results:**

Fifteen cases with ALK mutations and 57 cases without mutations were identified. The result of the one‐way ANOVA analysis showed only CEA was significantly associated with ALK mutations (95% CI:39.05‐148.88; *P* = .001). The area under the ROC curve (AUC) of CEA was 0.705 (95%CI:0.567‐0.843; *P* = .015). However, no significant association was observed between CEA and ALK mutations though the result of correlation analysis (*P* = .069) and multivariate logistic regression analysis (OR = 0.988, 95% CI: 0.972‐1.003, *P* = .111).

**Conclusions:**

In our study, we performed on the patients with stage IV lung adenocarcinoma in our region and found preoperative serum levels of SCCAg, CYRF21‐1, and NSE not suitable for the detection of ALK mutation. Although we observed a significant association between CEA and ALK mutations; however, it was not strong enough to distinguish ALK status for the patients in our region.

## 1 INTRODUCTION

Anaplastic Lymphoma Kinase (ALK) rearrangements are responsible for approximately 3%‐5% of non‐small cell lung cancer (NSCLC).^1^ Patients harboring ALK gene mutations benefit from ALK tyrosine kinase inhibitors (TKIs) treatment, which is a research hotspot concerning about targeted therapy for lung cancer in recent years.^2^ Currently, FDA‐approved ALK‐TKIs include the following: (a) crizotinib, the first generation of ALK‐TKI; (b) the second generation of ALK‐TKI, including ceritinib, alectinib, and brigatinib; and (c) the third generation of ALK‐TKI, such as Lorlatinib.^3^ These developments have prolonged the survival time of patients with advanced NSCLC, which led to the detection of ALK rearrangement as the standard of care for advanced NSCLC patients.

At present, there are three detection methods for ALK rearrangement, including real‐time polymerase chain reaction (RT‐PCR), immunohistochemistry (IHC), and fluorescence in situ hybridization(FISH).^4^ However, lacking of suitable sample for genetic testing in some cases restricts the detection of ALK rearrangement, which would limit the reasonable application of ALK‐TKI. Therefore, to analyze the characteristic findings of advanced NSCLC patients with ALK positive will help to distinguish ALK mutations.

Our previous work was to investigate the predictive power of 18F–FDG PET/CT for predicting positive ALK expression, which demonstrated that ALK‐positive patients tended to have a high the maximal standard uptake value (SUVmax) of lymph node.^5^ However, PET/CT examination is somewhat expensive and has still not become prevalent. Therefore, looking for an economical and convenient method to distinguish ALK mutations is promising. Blood examination as routine examination is the most widely used approach and its price is not high. So, we suggest that whether or not blood examination could be a valuable method for predicting the ALK rearrangement in NSCLC.

Carcinoembryonic antigen (CEA), squamous cell carcinoma antigen (SCCAg), CYFRA21‐1, and neuron‐specific enolase (NSE) served as the serum tumor markers have been widely used for the routine examination of NSCLC patients.^6^ In this study, we investigated the predictive value of tumor markers (CEA, SCCAg, CYFRA21‐1, and NSE) in the prediction of ALK rearrangements. There was a little similar study, which we would mention in discussion.

## 2 SUBJECTS AND METHODS

### 2.1 Study design

We retrospectively reviewed 482 cases with untreated lung adenocarcinoma during 2013‐2016 in Union Hospital, Tongji Medical College, Huazhong University of Science and Technology. Inclusion criteria include the following: (a) stage IV lung adenocarcinoma with ALK detection; (b) had no other malignancy; (c) had no received prior chemotherapy, radiotherapy or targeted therapy; (d) had tested for at least one of four serum tumor markers including CEA, SCCAg, CYFRA21‐1, and NSE before any anticancer therapy. We recorded the clinical data for patients, including age, gender, and the level of serum tumor markers (CEA, SCCAg, CYFRA21‐1, and NSE) before therapy.

### 2.2 Measurement of serum tumor marker levels

Blood samples collected before anticancer therapy were used for measurement of serum tumor marker levels including CEA, SCCAg, CYFRA21‐1, and NSE in the clinical laboratory of Wuhan Union Hospital. The concentrations of CEA, SCCAg, and CYFRA21‐1 were measured by chemiluminescence immunoassay. The cutoff values of CEA and SCCAg are 5.0 µg/L and 1.5 ng/mL, respectively. The cutoff values for CYFRA 21‐1 is 2.5 ng/mL. The level of NSE was detected by electrochemiluminescence immunoassay. The normal range of NSE level was determined as <16.3 µg/L.

### 2.3 ALK mutation analysis/

Mutational analysis of ALK was used Ventana IHC assay that described in our previous work.^5^ In brief, 4 μm‐thick formalin‐fixed, paraffin‐embedded tissue sections were stained by IHC that based on the monoclonal antibody, D5F3. The result was analyzed by pathologists from the Department of Pathology of Wuhan Union Hospital. Briefly, ALK positive was defined as the presence of any percentage of tumor cells with strong granular cytoplasmic staining.

### 2.4 Statistical analyses

The data of normal distribution were analyzed by independent samples t test, while the data of questionably normal distribution were analyzed by Mann‐Whiney U test. Point‐biserial correlation coefficient was calculated to test the relativity of serum tumor markers and the ALK gene mutation. The value of CEA in the prediction of mutation in the ALK oncogene was evaluated with receiver operating characteristic curve (ROC) curve. Multiple logistic regression analysis was used to assess the predictive power of preoperative serum tumor markers levels and missing data were supplemented by the method of the Expectation‐Maximization (EM) Algorithm. All analyses were performed by SPSS, v.22.0 (IBM), and *P* < .05 was considered statistically significant.

## 3 RESULTS

A total of 482 cases with untreated lung adenocarcinoma were retrospectively reviewed.

**Figure 1:**
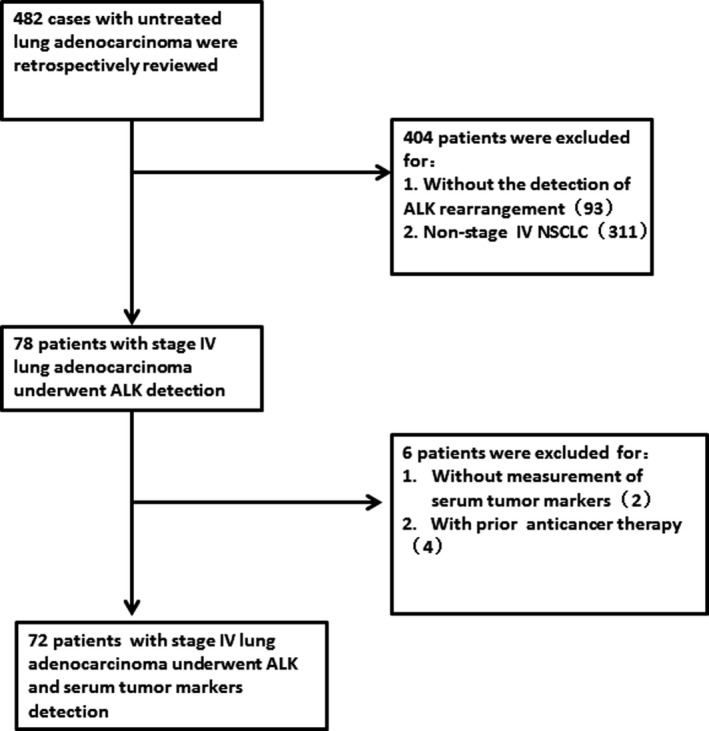
Selection process for patients in stage IV untreated lung adenocarcinoma with the detection of ALK mutations. Abbreviations: ALK, anaplastic lymphoma kinase; NSCLC, non‐small cell lung cancer

From these, 410 patients were excluded for the following reasons (Figure 1): 93 cases because they had no the detection of ALK rearrangement； 311 patients were not in the stage IV； 2 patients had no the detection of serum tumor markers; four patients recorded the levels of tumor markers after anticancer therapy. Finally, 72 patients (41 females and 31 males; age range, 29‐78 years; average age, 54.19 years) were included in this retrospective study. The level of serum tumor markers for these patients are shown in Table 1. 100% (72/72) of patients had CEA tested, 88% (63/72) of patients had SCCAg checked, 72% (52/72) of patients had CYFRA21‐1 recorded, and 79% (57/72) of patients had the detection of NSE. Fifteen cases mutations in the ALK gene and 57 patients in wild‐type ALK were detected in current study.

**Table 1:** Differences of clinical features in patients with or without ALK mutations

	ALK		*P*
	Mutant (n = 15)	Wild type (n = 57)	
CEA (μg/L)			
N	15(100%)	57(100%)	
Mean ± SD	21.73 ± 36.58	115.70 ± 195.10	.001**
95%CI			(39.05, 148.88)
SCCAg (ng/mL)			
N	13(87%)	50(88%)	
Mean ± SD	1.08 ± 0.55	1.21 ± 1.72	.778
95%CI			(−0.83, 1.11)
CYFRA21‐1 (ng/mL)			
N	11(73%)	41(72%)	
Mean ± SD	5.52 ± 7.07	9.92 ± 15.16	.356
95%CI			(−5.17, 13.22)
NSE (μg/L)			
N	13(87%)	44(77%)	
Mean ± SD	17.82 ± 6.62	19.18 ± 6.68	.521
95%CI			(−2.86, 5.58)

Abbreviations: ALK, anaplastic lymphoma kinase; CEA, carcinoembryonic antigen; NSE, neuron‐specific enolase; SCCAg, squamous cell carcinoma antigen.

** *P* value is less than. 01.

**Figure 2:**
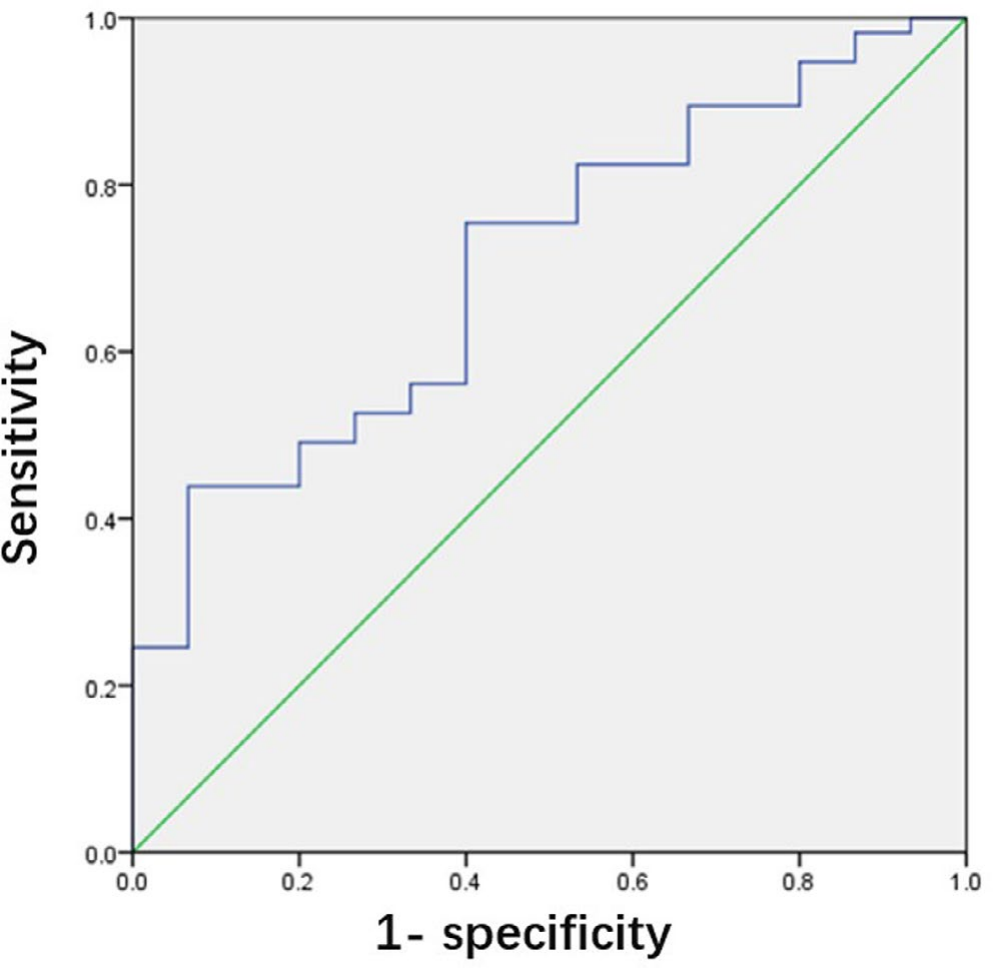
ROC curve for the predictive powers of CEA in the prediction of ALK mutations. Abbreviations: CEA, carcinoembryonic antigen; ROC, receiver operating characteristic curve

According to the analysis of Mann‐Whiney *U* test, a significant difference in CEA was found between the mutant ALK and wild‐type groups, which was lower in the mutant ALK groups (21.73 ± 36.58 vs 115.70 ± 195.10 *P* = .001; 95%CI:39.05‐148.88); however, no significant difference was observed in the level of SCCAg, CYFRA21‐1, and NSE between patients with and without ALK mutations. (*P* = .778, .356, .521, respectively) (Table1). We used ROC analysis to evaluate the predictive power of CEA in the prediction of ALK mutations. The area under the ROC curve (AUC) was 0.705 ± 0.015 (95%CI:0.567‐0.843; *P* = .015) (Table 2 and Figure2). However, the result of correlation analysis showed there was no significant association between CEA, SCCAg, CYFRA21‐1, and NSE (*P* = .069, .778, .384 and .521, respectively) (Table 3).

**Table 2:** ROC analysis to access the predictive power of CEA in the prediction of ALK mutations

	AUC	*P*	95%CI
CEA (μg/L)	0.705	.015*	(0.567‐0.843)

Abbreviations: ALK, anaplastic lymphoma kinase; CEA, carcinoembryonic antigen.

* *P* value is less than. 05.

**Table 3:** Correlation analysis of relationship between serum tumor markers and ALK Mutations

	Point‐biserial correlation coefficient	*P* value
CEA (μg/L)	−.216	.069
SCCAg (ng/ml)	−.036	.778
CYFRA21‐1 (ng/ml)	−.120	.384
NSE (μg/L)	−.087	.521

Abbreviations: ALK, anaplastic lymphoma kinase; CEA, carcinoembryonic antigen; NSE, neuron‐specific enolase; SCCAg, squamous cell carcinoma antigen.

**Figure 3:**
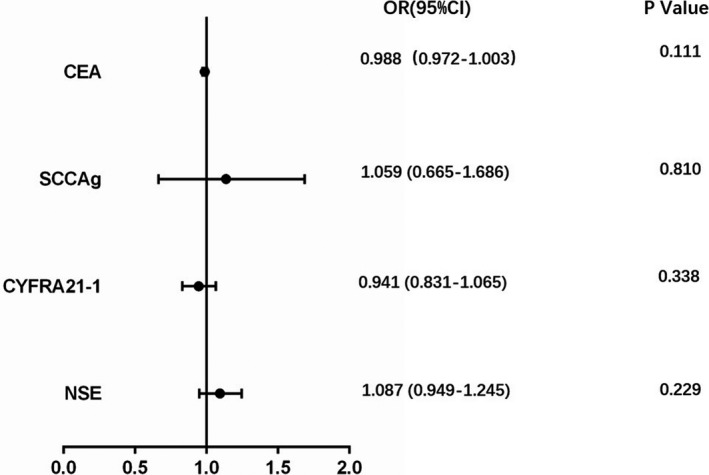
Multivariate analysis of relationship between between CEA (OR = 0.988, 95% CI: 0.972‐1.003, *P* = .111), SCCAg (OR = 1.059, 95% CI: 0.665‐1.686, *P* = .810), CYFRA21‐1 (OR = 0.941, 95% CI: 0.831‐1.065, *P* = .338), NSE (OR = 1.087, 95% CI: 0.949‐1.245, *P* = .229) and ALK mutations. Abbreviations: ALK, anaplastic lymphoma kinase; CEA, carcinoembryonic antigen; NSE, neuronspecific enolase; SCCAg, squamous cell carcinoma antigen

We then used correlation analysis and multivariate logistic regression analysis to further evaluate the relationship between serum tumor markers and ALK mutations. As described above, all patients in the study had detected the level of CEA without missing value. However, 88% (63/72) of patients had SCCAg checked, 72% (52/72) of patients had CYFRA21‐1 recorded, and 79% (57/72) of patients had the detection of NSE. The absent rate of serum tumor markers was as follows: SCCAg 12% (9/72), CYFRA21‐1 28% (20/72), and NSE 21% (15/72), respectively. We used the method of EM Algorithm to fill the missing value. On the foundation of missing value filling, the quality of data was evaluated and shown in Table 4. In ALK mutant group, the mean and standard deviation of SCCAg, CYFRA21‐1, and NSE had no significant change compared with the non‐supplemented group (1.09 ± 0.51 vs 1.08 ± 0.55; 5.73 ± 6.19 vs 5.52 ± 7.07; 17.97 ± 6.14 vs 17.82 ± 6.62, respectively). In ALK wild‐type group, there also was no significant change in SCCAg, CYFRA21‐1, and NSE after supplemented(1.21 ± 1.61 vs 1.21 ± 1.72; 9.46 ± 13.18 vs 9.92 ± 15.16; 19.87 ± 7.35 vs 19.18 ± 6.68, respectively).However, though the result of multivariate logistic regression analysis, no significant association was observed between CEA (OR = 0.988, 95% CI: 0.972‐1.003, *P* = .111), SCCAg(OR = 1.059, 95% CI: 0.665‐1.686, *P* = .810), CYFRA21‐1(OR = 0.941, 95% CI: 0.831‐1.065, *P* = .338), and NSE (OR = 1.087, 95% CI: 0.949‐1.245, *P* = .229)and ALK mutations (Figure3).

**Table 4:** Comparing the mean and standard deviation between non‐supplemented group and supplemented group

	SCCAg	CYFRA21‐1	NSE
ALK wild type			
Non‐supplemented			
Mean ± SD	1.21 ± 1.72	9.92 ± 15.16	19.18 ± 6.68
Supplemented			
Mean ± SD	1.21 ± 1.61	9.46 ± 13.18	19.87 ± 7.35
ALK mutant			
Non‐supplemented			
Mean ± SD	1.08 ± 0.55	5.52 ± 7.07	17.82 ± 6.62
Supplemented			
Mean ± SD	1.09 ± 0.51	5.73 ± 6.19	17.97 ± 6.14

Abbreviations: ALK, anaplastic lymphoma kinase; NSE, neuron‐specific enolase; SCCAg, squamous cell carcinoma antigen.

## 4 DISCUSSION

ALK gene rearrangements often mean that patients may be benefit from ALK‐TKIs treatment, which is glad tidings, especially for those with stage IV lung adenocarcinoma. So, the detection of ALK rearrangements play an important role in the targeted therapy. Considering the limitation of the obtainment of specimen, a lot of research were conducted to search a new method or index for predicting ALK mutations. Our previous work was to investigate the predictive power of parameters of PET/CT for the prediction of ALK mutations and found that a high‐level of SUVmax of lymph node related to ALK mutations significantly.^5^ However, the limitation of PET/CT includes low popularity and its high price. Therefore, we proposed research hypothesis for serum tumor markers, which is economical and convenient for every patient in the prediction of ALK mutations. We have investigated the relationship between serum tumor markers (CEA, SCCAg, CYFRA21‐1, and NSE) and ALK mutations in the patients in our region. No significant correlation was observed between serum tumor markers and ALK mutations except CEA (*P* = .015, AUC: 0.705); however, its predictive value was not a very strong.

CEA was one of clinical commonly used tumor markers and has been regarded as an assistant diagnostic index for NSCLC, especially for adenocarcinoma.^7^ Therefore, CEA has been studied to help predict the clinical features of the patients with NSCLC, including diagnosis and prognosis.^8^, ^9^ The study showed by Wen‐Tao Wang et al was the first study to research the association between CEA and ALK mutations, in their study, they reviewed retrospectively 92 lung adenocarcinoma patients and identified 8 patients who harbored the ALK mutations (8.7%, 8/92), which included 3 patients in the stage I and II and 5 patients in the stage III and IV. They found that the level serum CEA was significantly associated with ALK mutations in patients with lung adenocarcinoma.^10^ The similar result also observed in the study of Zeng Wang et al, in which they identified 20 patients with ALK mutations from 422 cases with lung adenocarcinoma in the stage I ~ III and found that CEA could predict independently ALK mutations.^11^ Recently, Jie Liu et al performed on 701 patients with advanced lung adenocarcinoma(stage Ⅲb and Ⅳ) and observed that CEA (OR = 0.481, 95% CI: 0.275‐0.842, *P* = .010) were independent predictors of ALK mutations.^12^ Similarly, in our current study, a significant difference between CEA and ALK mutations was overserved (95% CI:39.05‐148.88; *P* = .001). However, the predictive power of CEA was not a very strong, although its AUC was 0.705 (95% CI: 0.567‐0.843, *P* = .015), *P* value of correlation analysis and multivariate logistic regression analysis was .111 and .069, respectively. The reasons for the different results may be due to different research objects. In our study, we mainly study patients in the stage IV, whose lifesaving straw usually was targeted therapy. Because they are not eligible for surgical treatment, lacking of suitable sample for genetic testing occurred commonly. As the result, to research a suitable indicator to detect ALK mutations is very important for patients in the stage IV. An elevated level of CEA is associated with significantly the clinical stage IV ((*P* = .002), which was observed form the study performed by Sinead A. Noonan et al^13^ In another word, the predictive power of CEA was not very strong in our study, which was little different from the others may be due to removing the effect of clinical stage. However, the causes of difference cannot deny the contribution of population differences. In current study, we mainly focus on the patients in Hubei province, Central China. Furthermore, whether using CEA to detect ALK mutations is suitable for the population in our region are required larger studies to confirm, because through our strict inclusion criteria, the number of patients was small. Extending this finding would be our future research.

CYFRA21‐1, SCCAg, and NSE also were the three common serum tumor markers for NSCLC. Similarly, we investigated their predictive power in the detection of ALK mutations. Unfortunately, no significant correlation was observed between these tumor makers and ALK mutations. Our results were consistent with the previous reports.^10^, ^12^ It seems to suggest that CYFRA21‐1, SCCAg, and NSE are not suitable for the detection of ALK mutations, which need further studies to confirm.

However, we shouldn't neglect the limitation of our study. First, although we mainly studied the population of our region, our study is a single institutional study and the sample size was relatively small due to our strict inclusion criteria. Second, we were unable to control all factors which were associated with the level of tumor makers or ALK mutations, which may be due to introduced bias of retrospective design.

In summary, we studied on 72 patients in stage IV lung adenocarcinoma in Hubei province and investigated the predictive value of serum tumor markers in the prediction of ALK mutations, which we cannot predict through the level of tumor markers (SCCAg, CYRF21‐1, and NSE). Only the level of CEA was significantly associated with ALK mutations; however, It was not strong, which needs a large number of samples to extend these findings.

## References

[1] Soda M , Choi YL , Enomoto M , et al. Identification of the transforming EML4–ALK fusion gene in non‐small‐cell lung cancer. Nature. 2007;448:561. 17625570 10.1038/nature05945

[2] Iacono D , Chiari R , Metro G , et al. Future options for ALK ‐positive non‐small cell lung cancer. Lung Cancer. 2015;87:211‐219. 25601484 10.1016/j.lungcan.2014.12.017

[3] Chen L , Humphreys A , Turnbull L , et al. Identification of different ALK mutations in a pair of neuroblastoma cell lines established at diagnosis and relapse. Oncotarget. 2016;7:87301‐87311. 27888620 10.18632/oncotarget.13541 PMC5349989

[4] Wu Y‐C , Chang I‐C , Wang C‐L , et al. Comparison of IHC, FISH and RT‐PCR methods for detection of ALK rearrangements in 312 non‐small cell lung cancer patients in Taiwan. PLoS ONE. 2013;8:e70839‐e70839. 23951022 10.1371/journal.pone.0070839 PMC3737393

[5] Lv Z , Fan J , Xu J , et al. Value of (18)F‐FDG PET/CT for predicting EGFR mutations and positive ALK expression in patients with non‐small cell lung cancer: a retrospective analysis of 849 Chinese patients. Eur J Nucl Med Mol Imaging. 2018;45:735‐750. 29164298 10.1007/s00259-017-3885-z PMC5978918

[6] Molina R , Filella X , Auge JM , et al. Tumor markers (CEA, CA 125, CYFRA 21–1, SCC and NSE) in patients with non‐small cell lung cancer as an aid in histological diagnosis and prognosis. Comparison with the main clinical and pathological prognostic factors. Tumour Biol. 2003;24:209‐218. 14654716 10.1159/000074432

[7] Bergman B , Brezicka FT , Engström CP , Larsson S . Clinical usefulness of serum assays of neuron‐specific enolase, carcinoembryonic antigen and CA‐50 antigen in the diagnosis of lung cancer. Eur J Cancer. 1993;29:198‐202. 10.1016/0959-8049(93)90174-e 8380696

[8] Muley T , Rolny V , He Y , et al. The combination of the blood based tumor biomarkers cytokeratin 19 fragments (CYFRA 21–1) and carcinoembryonic antigen (CEA) as a potential predictor of benefit from adjuvant chemotherapy in early stage squamous cell carcinoma of the lung (SCC). Lung Cancer. 2018;120:46‐53. 29748014 10.1016/j.lungcan.2018.03.015

[9] Bai Y , Shen W , Zhu M , et al. Combined detection of estrogen and tumor markers is an important reference factor in the diagnosis and prognosis of lung cancer. J Cell Biochem. 2019;120(1):105‐114. 30216488 10.1002/jcb.27130

[10] Wen‐Tao W , Yin L , Jie M , Xiao‐Bing C , Jian‐Jun Q . Serum carcinoembryonic antigen levels before initial treatment are associated with EGFR mutations and EML4‐ ALK fusion gene in lung adenocarcinoma patients. Asian Pac J Cancer Prev. 2014;15:3927‐3932. 24935562 10.7314/apjcp.2014.15.9.3927

[11] Wang Z , Yang S , Lu H . Preoperative serum carcinoembryonic antigen levels are associated with histologic subtype, EGFR mutations, and ALK fusion in patients with completely resected lung adenocarcinoma. Onco Targets Ther. 2017;10:3345‐3351. 28744138 10.2147/OTT.S134452 PMC5511014

[12] Liu J , Zhao YQ , Han X , Hu XF , Wu HB , et al. Correlation between pre‐treatment serum carcinoembryonic antigen levels and genotypes in a large population of Chinese people with advanced lung adenocarcinoma. Intern Med J. 49(5):634‐643. 30379408 10.1111/imj.14152

[13] Noonan SA , Patil T , Gao D , et al. Brief Report: Baseline and on treatment characteristics of serum tumor markers in stage IV oncogene‐addicted adenocarcinoma of the lung. J Thorac Oncol. 2017;13:S1556086417306809. 10.1016/j.jtho.2017.08.005 PMC5810959 28843358

